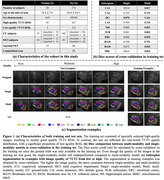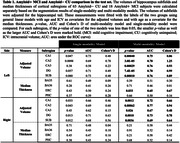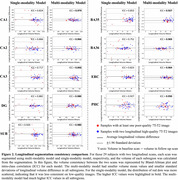# Robust multi‐modality segmentation of medial temporal lobe subregions using both 3‐tesla and 7‐tesla magnetic resonance imaging

**DOI:** 10.1002/alz.092878

**Published:** 2025-01-09

**Authors:** Yue Li, Pulkit Khandelwal, Long Xie, Christopher D. Brown, Xueying Lyu, Amanda E Denning, Mengjin Dong, Sandhitsu R. Das, David A Wolk, Paul A. Yushkevich

**Affiliations:** ^1^ University of Pennsylvania, Philadelphia, PA USA; ^2^ Siemens Healthineers, Princeton, NJ USA

## Abstract

**Background:**

Volumetry of subregions in the medial temporal lobe (MTL) computed from automatic segmentation in MRI can track neurodegeneration in Alzheimer’s disease. However, dropout artifacts are present in some modalities, leading to poor image quality and unreliable segmentation of MTL subregions. Considering that MRI modalities with different field strength offer distinct advantages in imaging different parts of the MTL, we developed a muti‐modality segmentation model using both 7‐tesla (7T) and 3‐tesla (3T) structural MRI to obtain robust segmentation in poor‐quality images.

**Method:**

MRI modalities including 3T T1‐weighted, 3T T2‐weighted, 7T T1‐weighted and 7T T2‐weighted (7T‐T2) of 197 subjects were collected from a longitudinal aging study at the Penn Alzheimer’s Disease Research Center. Among them, 7T‐T2 was used as the primary modality, and all other modalities were rigidly registered to the 7T‐T2. A model derived from nnU‐Net took these registered modalities as input and outputted subregion segmentation in 7T‐T2 space. High‐quality 7T‐T2 images from 25 selected training subjects were manually segmented to train the multi‐modality model. Modality augmentation, which replaced certain modalities with Gaussian noise randomly, was applied during training to guide the model to extract information from all modalities. To compare our proposed model with a baseline single‐modality model in the full dataset with mixed high/poor image quality, we evaluated the ability of derived volume/thickness measures to discriminate Amyloid+ mild cognitive impairment (A+MCI) and Amyloid‐ cognitively unimpaired (A‐CU) groups, as well as the stability of these measurements in longitudinal data.

**Result:**

Figure 1 shows characteristics of both training and test sets, as well as segmentation performance of models. The multi‐modality model delivered good performance regardless of 7T‐T2 quality, while the single‐modality model under‐segmented subregions in poor‐quality example images. The multi‐modality model generally demonstrated stronger discrimination of A+MCI versus A‐CU (Table 1). Intra‐class correlation and Bland‐Altman plots demonstrate that the multi‐modality model had higher longitudinal segmentation consistency in all subregions while the single‐modality model had low consistency in poor‐quality images (Figure 2).

**Conclusion:**

Using multi‐modality MRI, we provide an automatic MTL subregion segmentation algorithm that is robust to image quality. Our findings can help develop improved imaging biomarkers for neurodegeneration.